# Prevalence of spine surgery navigation techniques and availability in Africa: A cross-sectional study

**DOI:** 10.1016/j.amsu.2021.102637

**Published:** 2021-07-29

**Authors:** Ulrick Sidney Kanmounye, Yvan Zolo, Faith C. Robertson, Nourou Dine Adeniran Bankole, Kantenga Dieu Merci Kabulo, Jeff M. Ntalaja, Juma Magogo, Ahmed Negida, Nqobile Thango, Ignatius Esene, Brenton Pennicooke, Camilo A. Molina

**Affiliations:** a Research Department, Association of Future African Neurosurgeons, Yaounde, Cameroon; b Faculty of Health Sciences, University of Buea, Buea, Cameroon; c Department of Neurosurgery, Massachusetts General Hospital, Boston, MA, USA; d Neurosurgery Unit, Department of Surgery, Mohammed V University, Rabat, Morocco; e Neurosurgery Unit, Jason Sendwe Provincial Hospital, Lubumbashi, Democratic Republic of the Congo; f Neurosurgery Unit, Ngaliema General Provincial Reference Hospital, Kinshasa, Democratic Republic of the Congo; g Muhimbili Orthopedic Institute, Dar es Salaam, Tanzania; h Faculty of Medicine, Zagazig University, Zagazig, El-Sharkia, Egypt; i Division of Neurosurgery, Department of Surgery, University of Cape Town, Cape Town, South Africa; j Neurosurgery Division, Faculty of Health Sciences, University of Bamenda, Bambili, Cameroon; k Department of Neurosurgery, Washington University School of Medicine, St. Louis, Missouri, USA

**Keywords:** Access, Africa, Computer-assisted surgery, Neuronavigation, Spine surgery

## Abstract

**Background:**

Africa has a large burden of spine pathology but has limited and insufficient infrastructure to manage these spine disorders. Therefore, we conducted this e-survey to assess the prevalence and identify the determinants of the availability of spine surgery navigation techniques in Africa.

**Materials and methods:**

A two-part questionnaire was disseminated amongst African neurological and orthopedic surgery consultants and trainees from January 24 to February 23, 2021. The Chi-Square, Fisher Exact, and Kruskal-Wallis tests were used to evaluate bivariable relationships, and a p-value <0.05 was considered statistically significant.

**Results:**

We had 113 respondents from all regions of Africa. Most (86.7 %) participants who practiced or trained in public centers and centers had an annual median spine case surgery volume of 200 (IQR = 190) interventions. Fluoroscopy was the most prevalent spine surgery navigation technique (96.5 %), followed by freehand (55.8 %), stereotactic without intraoperative CT scan (31.9 %), robotic with intraoperative CT scan (29.2 %), stereotactic with intraoperative CT scan (8.8 %), and robotic without intraoperative CT scan (6.2 %). Cost of equipment (94.7 %), lack of trained staff to service (63.7 %), or run the equipment (60.2 %) were the most common barriers to the availability of spine instrumentation navigation. In addition, there were significant regional differences in access to trained staff to run and service the equipment (P = 0.001).

**Conclusion:**

There is a need to increase access to more advanced navigation techniques, and we identified the determinants of availability.

## Highlights

• African spine surgery has regional disparities in navigation techniques.
• Fluoroscopy is the most prevalent navigation technique.
• Cost and lack of trained personnel are the most significant barriers.

## 1 Introduction

Spine disorders affect about 50 million Africans each year, and almost 200 000 require neurosurgical management [1,2]. Although the African continent has the lowest prevalence of diagnosed spine disorders, much of the surgical disease remains untreated given the lack of resources to provide operative management [[2], [3], [4]]. One area in which Africa has a deficit is in infrastructure and equipment.

Over the past three decades, spine surgeons have improved the quality of spine care, and these advancements are in part due to innovations within the field of image-guided spine surgery [5]. The use of intraoperative CT, stereotaxy, and robotics significantly reduces screw malposition compared to freehand and fluoroscopic screw placement [[6], [7], [8]]. Intraoperative CT, stereotaxy, and robotics are even more valuable in complex spine and cervical spine cases as they afford better appreciation of surrounding neural and vascular structures and ultimately better surgical planning [[9], [10], [11], [12]].

Despite having better outcomes, access to spine neuronavigation techniques is often limited by cost. The average cost of a navigation system is USD 475 000, and this is compounded by the necessity to have trained personnel for the system's manipulation and preventive maintenance [13,14]. It has been suggested that these costs can be offset with reduced operative time and reoperation rates [[15], [16], [17]]. While this assertion might be valid in North America and Europe, it is rarely true in Africa. Patients often present late, do not have health coverage, and centers have competing priorities [2,18]. In addition, these expensive equipment are often operated in unfavorable conditions (ex: humidity and unstable power supply, and with limited access to skilled biomedical engineers and after-sales services) [19].

Most African centers have access to CT scans (97.3 %) and MRIs (78.6 %) outside the operative room [20]. However, little is known about the availability of spine surgery navigation in Africa. In this e-survey, the authors sought to map the prevalence of spine surgery navigation techniques in Africa and identify determinants of their availability.

## 2 Materials and Methods

The study was registered to ClinicalTrials.gov Protocol Registration and Results System (Registration ID: NCT04927273; https://clinicaltrials.gov/ct2/show/NCT04927273?term=NCT04927273&draw=2&rank=1).

The authors followed the STrengthening the Reporting of OBservational studies in Epidemiology (STROBE) and STrengthening the Reporting Of Cohort Studies in Surgery (STROCSS) statement guidelines when reporting this manuscript [21,22].

### 2.1 Study design, setting, and duration

From January 24 to February 23, 2021, we conducted a cross-sectional study using an online survey distributed among African neurosurgeons and orthopedic surgeons involved in spine surgeries.

### 2.2 Study variables and survey development

We developed a two-part questionnaire in English to collect data on the prevalence of spine surgery navigation techniques in Africa. The questions were validated by the senior author (CM), a spine surgeon at a large US academic center, and two African spine surgeons with more than ten years of experience. The questionnaire was piloted among ten residents and neurosurgeons to ensure usability and technical functionality. The responses from the pilot were not included in the final analyses. The first part of the questionnaire had seven questions and collected sociodemographic data (sex, age, specialty, academic level, country of practice, type of hospital, and mean annual spine surgery case volume). The second part of the questionnaire had eight questions that collected data on the availability and barriers to spine surgery navigation. These questions were disaggregated by spine level (occipital and axial cervical, subaxial, thoracic, lumbosacral, and pelvic) (**Supplemental Material 1, Survey Questionnaire**). Respondents working in multiple centers were prompted to give responses for each center.

### 2.3 Data collection and sampling methods

The e-survey was hosted on Google Forms (Google, CA, USA) and distributed via social media (WhatsApp and Facebook; Facebook Inc. California, USA) to African neurosurgery and orthopedic residents and consultants. The e-survey link was shared daily on these social media platforms for one month (January 24 to February 23, 2021). Participants were recruited using convenient sampling. Due to the wide dissemination of the questionnaire through social media platforms, calculation of a response rate was not possible; 95 % confidence intervals have been used and documented as (%-%) after the figures in Tables A1, A.2, and A.3.

**Table 1:** Sociodemographic characteristics. 95 % confidence intervals are shown in Supplemental Material 2.

Characteristics	Frequency (Percentage)
Sex	
Male	95 (84.1)
Female	18 (15.9)
Country	
Egypt	26 (23.0)
Morocco	25 (22.1)
Ivory Coast	16 (14.2)
Zimbabwe	8 (7.1)
Democratic Republic of the Congo	7 (6.2)
Nigeria	7 (6.2)
Tanzania	7 (6.2)
Cameroon	5 (4.4)
Kenya	4 (3.5)
Libya	2 (1.8)
Mozambique	2 (1.8)
Malawi	1 (0.9)
Mali	1 (0.9)
Namibia	1 (0.9)
South Africa	1 (0.9)
Specialty	
Neurosurgery	98 (86.7)
Orthopedics	15 (13.3)
Practice	
Public academic	84 (74.3)
Private	22 (19.5)
Public non-academic	15 (3.3)
Military	10 (8.8)
Academic level	
Resident	50 (44.2)
Consultant	47 (41.6)
Fellow	16 (14.2)

### 2.4 Ethics

Participation was voluntary, and no financial incentivization was involved. The respondents' consents were sought at the beginning of the survey, and they were permitted to discontinue or decline to answer a question whenever they chose. The survey data were stored in a password-protected account, and access to the data was limited to the authors. The institutional review board of the Bel Campus University of Technology issued an ethics waiver.

### 2.5 Statistical analysis

Continuous variables as age were summarized as mean (standard deviation) or median (interquartile ranges) for normally and non-normally distributed data, respectively. Data normality was tested by the Kolmogorov Smirnov test.

The authors calculated the respondents' mean age with its standard deviation and the median annual spine surgical volume with its interquartile range. All qualitative sociodemographic and spine surgery navigation availability data were expressed as frequencies and percentages. The Kruskal Wallis test was used to compare the annual spine surgery surgical volume between centers. The association between the availability of spine surgery navigation techniques and relevant independent variables (specialty, type of hospital, and barriers to spine surgery navigation techniques) was evaluated using the Chi-Square or Fisher's Exact tests. A P-value <0.05 was considered statistically significant. The odds ratio and its 95 % confidence interval were equally reported. Next, statistically significant variables were included in the binomial regression analyses.

## 3 Results

### 3.1 Participants' characteristics

One hundred thirteen orthopedic or neurological surgery consultants and trainees from 15 African countries responded to the e-survey. They were 37.3 ± 8.9 years old and most were male (n = 95, 84.1 %), practicing or training in the neurosurgery units (n = 98, 86.7 %) of public academic centers (n = 84, 74.3 %) (Table 1).

### 3.2 Spine surgery case volume

**Fig. 1:**
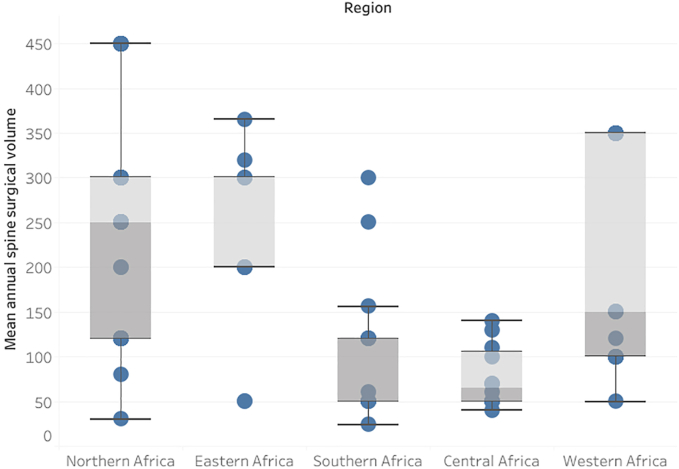
Box plot of the regional mean annual spine surgical case volumes.

The centers had an annual median spine case surgery volume of 200 (IQR = 190) interventions. Western African centers had the highest surgical case volumes (median = 350.0; IQR = 200.0) followed by Northern (median = 250.0; IQR = 180.0), Eastern (median = 200.0; IQR = 100.0), Southern (median = 120.0; IQR = 88.0), and Central (median = 65.0; IQR = 58.0) African centers (P < 0.001) (Fig. 1). Public academic centers had 261.2 ± 226.7, private centers had 250.5 ± 124.7, military centers had 245.3 ± 205.0, and public non-academic centers had 178.1 ± 107.4 mean annual spine cases (P < 0.001). Neurosurgery spine centers had greater mean annual operative volumes than orthopedic centers (254.1 ± 214.0 vs. 187.3 ± 121.6; P = 0.24).

### 3.3 Availability and barriers to spine surgery navigation techniques

**Fig. 2:**
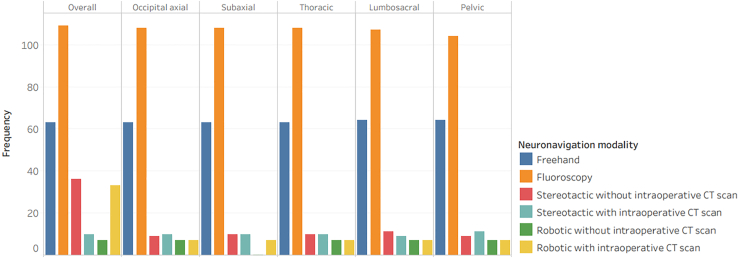
Availability of spine instrumentation navigation. 95 % confidence intervals are shown in Supplemental Material 2.

Fluoroscopy was the most prevalent spine surgery navigation technique (n = 109, 96.5 %) followed by freehand (n = 63, 55.8 %), stereotactic without intraoperative CT scan (n = 36, 31.9 %), robotic with intraoperative CT scan (n = 33, 29.2 %), stereotactic with intraoperative CT scan (n = 10, 8.8 %), and robotic without intraoperative CT scan (n = 7, 6.2 %). Fig. 2 illustrates the overall and segmental availability of spine instrumentation navigation.

**Fig. 3:**
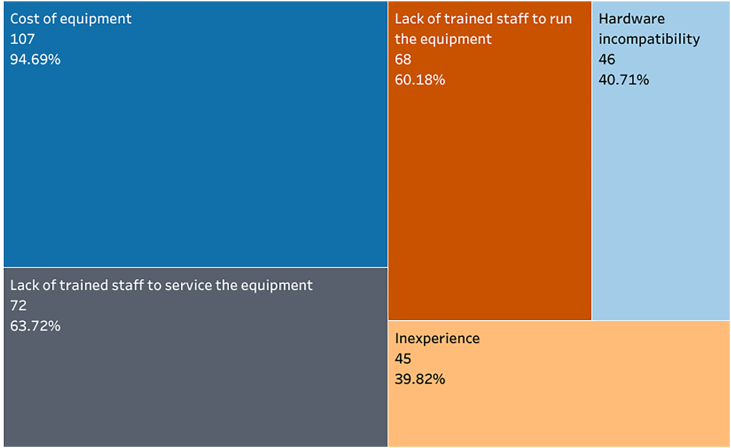
TreeMap of the barriers to the availability of spine instrumentation navigation. 95 % confidence intervals are shown in Supplemental Material 2.

The majority (n = 111, 98.2 %) of respondents reported barriers to the availability of spine instrumentation navigation: 27 (23.9 %) reported facing a single barrier, 13 (11.5 %) faced two, 34 (30.1 %) faced three, 2 (1.8 %) faced four, and 35 (31.0 %) faced five barriers. Cost of equipment (n = 107, 94.7 %), lack of trained staff to service the equipment (n = 73, 63.7 %), and lack of trained staff to run the equipment (n = 68, 60.2 %) were the most common barriers to the availability of spine instrumentation navigation (Fig. 3).

### 3.4 Factors influencing the availability of spine surgery instrumentation navigation

Central African respondents used freehand more often than other respondents (91.7 %, P < 0.001), and they all had access to fluoroscopy (100 %, P = 0.05). Stereotactic without CT and robotic with CT were more prevalent in Northern Africa (49.1 %, P = 0.002 and P < 0.001), while stereotactic with CT and robotic without CT were more prevalent in Western Africa (33.3 % and 29.2 %; P < 0.001) (Table 2). More Eastern African respondents reported hardware incompatibility and inexperience (63.6 %, P = 0.001) as barriers to the accessibility of spine surgery neuronavigation techniques. In comparison, more Southern African respondents reported a lack of trained staff to service and run the equipment (92.3 %, P = 0.001) (Table 3).

**Table 2:** Regional availability of spine surgery instrumentation navigation techniques. 95 % confidence intervals are shown in Supplemental Material 2.

Neuronavigation techniques	Northern Africa	Western Africa	Central Africa	Eastern Africa	Southern Africa	P-Value
Freehand	50.9 %	87.5 %	91.7 %	18.2 %	15.4 %	<0.001
Fluoroscopy	98.1 %	100 %	100 %	81.8 %	92.3 %	0.05
Stereotactic without CT	49.1 %	29.2 %	0 %	18.2 %	7.7 %	0.002
Stereotactic with CT	0 %	33.3 %	0 %	18.2 %	0 %	<0.001
Robotic without CT	0 %	29.2 %	0 %	0 %	0 %	<0.001
Robotic with CT	49.1 %	29.2 %	0 %	0 %	0 %	<0.001

**Table 3:** Regional barriers to spine surgery instrumentation navigation techniques. 95 % confidence intervals are shown in Supplemental Material 2.

Barriers	Northern Africa	Western Africa	Central Africa	Eastern Africa	Southern Africa	P-Value
Cost of equipment	94.2 %	95.8 %	91.7 %	100 %	100 %	0.78
Hardware incompatibility	56.6 %	12.5 %	25.0 %	63.6 %	23.1 %	0.001
Inexperience	54.7 %	12.5 %	25.0 %	63.6 %	23.1 %	0.001
Lack of trained staff to run the equipment	58.5 %	29.2 %	75.0 %	81.8 %	92.3 %	0.001
Lack of trained staff to service the equipment	69.8 %	33.3 %	50.0 %	81.8 %	92.3 %	0.001

Orthopedic surgeons were less likely to use freehand overall (26.7 % orthopedic surgery vs. 60.2 % neurosurgeons; OR = 0.24; 95 % CI = 0.07–0.81; P = 0.02) but they were more likely to use freehand for occipital and axial cervical cases, subaxial cervical cases, and for thoracic cases (80.0 % orthopedic surgery vs. 52.0 % neurosurgeons; OR = 3.69; 95 % CI = 0.98–13.88; P = 0.04). They faced two barriers more frequently than neurosurgeons: hardware incompatibility (73.3 % orthopedic surgeon vs. 35.7 % neurosurgeons; OR = 4.95; 95 % CI = 1.47–16.71; P = 0.01) and inexperience (73.3 % orthopedic surgeons vs. 34.7 % neurosurgeons; OR = 5.18; 95 % CI = 1.53–17.49; P = 0.004).

Private centers had no intraoperative CT scan (0.0 % private vs. 40.7 % non-private; OR = 0.59; 95 % CI = 0.50–0.70; P < 0.001), stereotactic guidance without intraoperative CT scan (0.0 % private vs. 39.6 % non-private; OR = 0.60; 95 % CI = 0.51–0.71; P < 0.001), or robotic with intraoperative CT scan (0.0 % private vs. 36.3 % non-private; OR = 0.64; 95 % CI = 0.55–0.74; P = 0.001). Respondents working at private centers were more likely to use freehand overall (77.3 % private vs. 50.5 % non-private; OR = 3.33; 95 % CI = 1.13–9.78; P = 0.02) but less likely to use freehand for occipital and axial cervical cases and for subaxial cervical cases (48.8 % private vs. 75.9 % non-private; OR = 0.30; 95 % CI = 0.12–0.79; P = 0.01). Private centers were less likely to face difficulties with hardware compatibility (34.5 % private vs. 58.6 % non-private; OR = 0.37; 95 % CI = 0.16–0.88; P = 0.03).

Public non-academic centers were less likely to have intraoperative CT scans (6.7 % public non-academic vs. 36.7 % not public non-academic; OR = 0.12; 95 % CI = 0.02–0.98; P = 0.02), robotic with intraoperative CT scan (0.0 % Public non-academic vs. 33.7 % Not public non-academic; OR = 0.66; 95 % CI = 0.58–0.76; P = 0.01) and stereotactic without intraoperative CT scan: 0.0 % Public non-academic vs. 36.7 % Not public non-academic; OR = 0.63; 95 % CI = 0.54–0.74; P = 0.004). Those working at these centers were more likely to use freehand for occipital and axial cervical cases, subaxial cervical, thoracic, lumbosacral, and pelvic cases (93.3 % public non-academic vs. 50.0 % not public non-academic; OR = 14.00; 95 % CI = 1.78–110.62; P = 0.002). In addition, they were less likely to use fluoroscopy for occipital and axial cervical cases (80.0 % public non-academic vs. 98.0 % not public non-academic; OR = 0.08; 95 % CI = 0.01–0.55; P = 0.002) and for subaxial cervical, thoracic, lumbosacral, and pelvic cases (73.3 % public non-academic vs. 94.9 % not public non-academic; OR = 0.15; 95 % CI = 0.03–0.63; P = 0.004). Public non-academic centers were more likely to lack trained staff to run their equipment (93.3 % public non-academic vs. 55.1 % not public non-academic; OR = 11.41; 95 % CI = 1.44–90.17; P = 0.01).

Participants practicing at academic centers were less likely to use freehand for thoracic cases (48.8 % academic vs. 75.9 % non-academic; OR = 0.30; 95 % CI = 0.12–0.79; P = 0.01) and for lumbosacral and pelvic cases (50.0 % academic vs. 75.9 % non-academic; OR = 0.32; 95 % CI = 0.12–0.82; P = 0.02). However, they were more likely to use fluoroscopy for pelvic cases (95.2 % academic vs. 82.8 % non-academic; OR = 4.12; 95 % CI = 1.04–16.76; P = 0.04). Lack of trained staff to run the equipment (52.4 % academic vs. 82.8 % non-academic; OR = 0.23; 95 % CI = 0.08–0.66; P = 0.004) and lack of trained staff to service the equipment (57.1 % academic vs. 82.8 % non-academic; OR = 0.28; 95 % CI = 0.10–0.80; P = 0.01) were less common in academic centers.

Participants who did not have fluoroscopy for occipital and axial cervical, subaxial cervical, and thoracic cases at their centers were more likely to report high costs of equipment as a barrier (OR = 23.11; 95 % CI = 2.76–193.64; P = 0.02). Similarly, high costs of equipment decreased the availability of fluoroscopy for lumbosacral (OR = 17.17; 95 % CI = 2.21–133.23; P = 0.02) and pelvic cases (OR = 25.25; 95 % CI = 3.52–180.99; P = 0.03). Centers that lacked intraoperative CT scan (OR = 0.34; 95 % CI = 0.14–0.81; P = 0.01), stereotactic without intraoperative CT scan (OR = 0.36; 95 % CI = 0.15–0.87; P = 0.02), stereotactic with intraoperative CT scan (OR = 0.85; 95 % CI = 0.77–0.94; P = 0.01) and robotics without intraoperative CT scan (OR = 0.90; 95 % CI = 0.83–0.97; P = 0.04) were less likely to report hardware incompatibility as a barrier. Inexperience was not a barrier to the availability of intraoperative CT scan (OR = 0.36; 95 % CI = 0.15–0.86; P = 0.02) or robotics (OR = 0.90; 95 % CI = 0.83–0.97; P = 0.03).

## 4 Discussion

In this e-survey, we investigated the prevalence and barriers to spine surgery navigation techniques in Africa. Respondents were from all regions of Africa (Northern, Western, Central, Eastern, and Southern), specialties (orthopedic surgery and neurosurgery), academic levels (residents, fellows, and consultants), and hospitals (public academic, public non-academic, private, and military). Fluoroscopy was the most prevalent spine surgery navigation technique, while robotic was the least prevalent. Cost of equipment and lack of personnel to operate and maintain equipment were the most common barriers to spine surgery navigation techniques.

Although widely reported by Central African respondents, equipment cost was not found to be a statistically significant barrier. However, the lack of trained staff to service and run the equipment were statistically significant barriers. Few centers in regions with greater access to advanced neuronavigation (i.e., Western and Northern Africa) reported a lack of trained staff as a barrier to the availability of neuronavigation. These findings support the widely held view that access to trained clinical engineers, biomedical engineers, and radiology technologists strengthens components of the surgical system (workforce, service delivery, and infrastructure) and improves patient outcomes [23]. Newly purchased equipment is more likely to break down due to suboptimal utilization, periodic preventive maintenance, and adverse tropical conditions (tropical storms, humidity, heat, and irregular power supplies) [[24], [25], [26]]. Manufacturers can help reduce these costs by offering capacity-building of operating personnel and biomedical engineers at African hospitals and designing tropicalized navigation systems. As of 2018, Africa had about 2000 clinical and biomedical engineers and more than 6425 biomedical technicians [27]. These numbers are increasing as a result of the growth of degree-granting biomedical engineering programs [28]. Hence, we anticipate that the impact of lack of trained personnel on the availability of spine neuronavigation techniques should be lessened in upcoming years. Future studies should investigate the geographical distribution and familiarity with neuronavigation of African clinical engineers, biomedical engineers, and radiology technologists. These investigations should improve our understanding of the lack of trained staff.

A total of 31.9 % of respondents had access to stereotactic without intraoperative CT scan, 8.8 % had access to stereotactic with intraoperative CT scan, 29.2 % had access to robotic with intraoperative CT scan, and 6.2 % had access to robotic without intraoperative CT scan. These findings can be explained by the fact that most computer navigation methods in Africa use registration methods such as surface matching methods to register the patient's anatomy to the pre-operative scan, and have limited access to pre-operative fluoroscopy [20]. In a 2013 survey of 677 spine surgeons worldwide, Härtl et al. [29] found 38 % of surgeons had access to computer-assisted navigation: 70 % in North America, 42 % in Europe, 42 % in Asia Pacific, and 14 % in Latin America. Of note, there were no African surgeons among the respondents of the worldwide survey. In another global survey, 60.3 % of young neurosurgeons had access to an image guidance system [30]. Only 24.1 % of young neurosurgeons have access to an image guidance system for cranial or spine surgery in Africa [20]. Our findings are similar to the African investigations [20,30], supporting that the prevalence of spine neuronavigation is lower in Africa than in North America, Europe, and Asia Pacific.

We found no evidence of a difference in the availability of neuronavigation between orthopedic surgeons and neurosurgeons. However, orthopedic surgeons were more likely to face problems related to hardware incompatibility and inexperience. In a survey of 306 spine surgeons in Latin America, Guiroy et al. [31] did not find evidence of a difference in access to neuronavigation between orthopedic surgeons and neurosurgeons. However, Härtl et al. [29] found that orthopedic surgeons were less likely to have access to neuronavigation (OR = 0.6; 95 % CI = 0.39–0.90; P = 0.02) [29].

In Härtl et al.'s series [29], the number of minimally invasive spine surgery cases was inversely proportional to the use of neuronavigation (OR = 1.7; 95 % CI = 1.1–2.5; P = 0.02). We found that public, academic centers had the largest spine surgery operating volumes, and neurosurgery centers had greater mean annual operative volumes than orthopedic centers. Public non-academic centers, the centers with the smallest operative volumes, were less likely to have intraoperative CT scan, stereotactic without intraoperative CT scan, and robotic with intraoperative CT scan. Similarly, private centers were less likely to have intraoperative CT scans, stereotactic without intraoperative CT scans, and robotic with intraoperative CT scans. Of note, private centers had the second-largest operative volume. Whereas the difference in the different types of hospitals' operative volumes was statistically significant, we found no evidence to support the surgical volume difference between both specialties.

The limitations of this study include issues related to convenience sampling methodologies that precluded response rate calculation, as well as the likely omission of responses from those without reliable internet or without electronic devices. Also, dissemination via social media is prone to sampling error, especially multiple responses from surveyees. We minimized this error by using Google Forms' limited responses feature, which uses email accounts as identifiers. Although it is useful, this method is ineffective against multiple response submissions from individuals with multiple email accounts. In addition, administering the survey in English limited respondents to those with sufficient English comprehension. Also, there are reports of task-shifting and -sharing in African spine surgery. In some underserved regions, general surgeons perform essential and emergency spine surgery. We did not capture this population in our survey.

This study expands the literature by providing information about the availability of spine surgery navigation techniques in Africa. Since Africa suffers a shortage in neurosurgical capacity and equipment, examining the availability of spine surgery navigation is important to map and guide future neurosurgery resource allocation efforts in Africa. The stereotactic with CT, robotic without CT, and robotic with CT techniques were not available in most of the surveyed centers, with the expensive cost of the equipment being the major barrier towards the availability of these equipment followed by the lack of trained staff to work on the equipment. This information is important for health policy decision-makers to consider allocating sufficient funds to provide this equipment and to provide staff and trainees with sufficient training on this equipment.

## 5 Conclusions

Africa counts a decent number of centers equipped with spine navigation technologies. In these centers, fluoroscopy is the most common spine navigation technology, and there is a need to increase the availability of other spine navigation modalities such as intraoperative CT scans, stereotactic and robotic spine navigation technologies. It is important to note that while increasing these spine navigation technologies on the continent, there is a need to equitably distribute them in all regions to permit patients to benefit from these technologies no matter their location. We identified that qualified human resources were the major determinant of the availability of spine surgery navigation techniques on the continent. Therefore, increasing the neurosurgical and orthopedic workforce with knowledge on how to operate spine navigation technologies will go a long way to densify the availability of these technologies in Africa. Moreover, frequently training this human resource on the latest spine navigation technology updates will also permit them to offer better services to the patients benefiting from these services.

## Provenance and peer review

Not commissioned, externally peer-reviewed.

## Ethical approval

Ethical approval was obtained from the institutional review board of Bel Campus University of Technology Ref: CEI/PCA/2020A345D64.

## Funding

The authors received no financial support for the research, authorship, and/or publication of this article.

## Author contribution

USK conceptualized the study, investigated, curated, analyzed, and visualized the data, wrote the original draft of the manuscript, and administered the project. CM supervised, administered the project, validated, and wrote the original manuscript draft. YZ, FCR, NDAB, KDMK, JNM, JM, AN, NT, IE, and BP investigated, validated, and wrote the original manuscript draft. All authors have read and approved the manuscript.

## Declaration of conflicting interests

The authors declare that there is no conflict of interest.

## Guarantor

Ulrick Sidney Kanmounye.
