# Supplementary material for: Prevalence of spine surgery navigation techniques and availability in Africa: A cross-sectional study
Source: Ann Med Surg (Lond). 2021 Jul 29;68:102637. doi: 10.1016/j.amsu.2021.102637 (PMC8346523; doi:10.1016/j.amsu.2021.102637)
Supplement: Multimedia component 2 [file mmc2.pdf]

Prevalence of spine surgery navigation techniques and availability in Africa

General Information

The aim of this study is to collect data on the availability and navigation techniques used for spine surgery in Africa. We appreciate your interest in this study. This should take 5 minutes on average.

If you have questions please contact Ulrick Sidney Kanmounye (ulrick.kanmounye@medadvisor-operationsmile.org)

*Required

Sociodemographic Characteristics

1. Which country do you work in? *

Mark only one oval.

- Angola
- Benin
- Botswana
- Burkina Faso
- Burundi
- Cameroon
- Cape Verde
- Central African Republic
- Chad
- Comoros
- Democratic Republic of the Congo
- Djibouti
- Egypt
- Equatorial Guinea
- Eritrea
- Eswatini
- Ethiopia
- Gabon
- Gambia
- Ghana
- Guinea
- Guinea-Bissau
- Ivory Coast
- Kenya
- Lesotho
- Liberia
- Libya
- Madagascar
- Malawi
- Mali

- Mali
- Mauritania
- Mauritius
- Morocco
- Mozambique
- Namibia
- Niger
- Nigeria
- Republic of the Congo
- Rwanda
- São Tomé and Príncipe
- Senegal
- Seychelles
- Sierra Leone
- Somalia
- South Africa
- South Sudan
- Sudan
- Tanzania
- Togo
- Tunisia
- Uganda
- Western Sahara
- Zambia
- Zimbabwe

2. Where do you practice? (select all that apply) *

Tick all that apply.

- [ ] Academic center
- [ ] Private center
- [ ] Public non-academic center
- [ ] Military hospital

Other: [ ] ______________________

3. What is the mean annual number of spine interventions (surgical volume) at your center *

____

4. Sex *

Mark only one oval.

( ) Female
( ) Male
( ) Prefer not to say
( ) Other: ______________________

5. Age *

Mark only one oval.

- 22
- 23
- 24
- 25
- 26
- 27
- 28
- 29
- 30
- 31
- 32
- 33
- 34
- 35
- 36
- 37
- 38
- 39
- 40
- 41
- 42
- 43
- 44
- 45
- 46
- 47
- 48
- 49
- 50
- 51

[illegible]

52

53

54

55

56

57

58

59

60

61

62

63

64

65

66

67

68

69

70

71

72

73

74

75

76

77

78

79

80

81+

6. Specialty *

Mark only one oval.

- Neurosurgery
- Orthopedics

7. Current role *

Mark only one oval.

- Resident
- Fellow/Subspecialty trainee
- Consultant/Attending surgeon

Availability of spine surgery navigation

8. Which of the following do you have access to at your center? *

Tick all that apply.

- [ ] Fluoroscopy (X-ray)
- [ ] Intraoperative CT Scan
- [ ] Robotics

9. What type of intraoperative instrumentation guidance do you have available? *

Tick all that apply.

- [ ] Freehand without flouroscopy
- [ ] Fluoroscopy
- [ ] Manual stereotactic computer navigation with intraop CT
- [ ] Manual stereotactic computer navigation without intraop CT using alternate registration methods
- [ ] Robotic navigation with intraop CT
- [ ] Robotic navigation without intraop CT using alternate registration methods

Other: [ ] ____________________

10. What type of instrumentation method do you use for the occipital and axial cervical spine? *

Tick all that apply.

- [ ] Freehand without flouroscopy
- [ ] Fluoroscopy
- [ ] Manual stereotactic computer navigation with intraop CT
- [ ] Manual stereotactic computer navigation without intraop CT using alternate registration methods
- [ ] Robotic navigation with intraop CT
- [ ] Robotic navigation without intraop CT using alternate registration methods

Other: [ ] ____________________

11. What type of instrumentation method do you use for the subaxial cervical spine? *

Tick all that apply.

- [ ] Freehand without flouroscopy
- [ ] Fluoroscopy
- [ ] Manual stereotactic computer navigation with intraop CT
- [ ] Manual stereotactic computer navigation without intraop CT using alternate registration methods
- [ ] Robotic navigation with intraop CT
- [ ] Robotic navigation without intraop CT using alternate registration methods

12. What type of instrumentation method do you use for the thoracic spine? *

Tick all that apply.

- [ ] Freehand without flouroscopy
- [ ] Fluoroscopy
- [ ] Manual stereotactic computer navigation with intraop CT
- [ ] Manual stereotactic computer navigation without intraop CT using alternate registration methods
- [ ] Robotic navigation with intraop CT
- [ ] Robotic navigation without intraop CT using alternate registration methods

Other: [ ] ____________________

13. What type of instrumentation method do you use for the lumbosacral spine? *

Tick all that apply.

- [ ] Freehand without flouroscopy
- [ ] Fluoroscopy
- [ ] Manual stereotactic computer navigation with intraop CT
- [ ] Manual stereotactic computer navigation without intraop CT using alternate registration methods
- [ ] Robotic navigation with intraop CT
- [ ] Robotic navigation without intraop CT using alternate registration methods

Other: [ ] ____________________

14. What type of instrumentation method do you use for the pelvis spine? *

Tick all that apply.

- [ ] Freehand without flouroscopy
- [ ] Fluoroscopy
- [ ] Manual stereotactic computer navigation with intraop CT
- [ ] Manual stereotactic computer navigation without intraop CT using alternate registration methods
- [ ] Robotic navigation with intraop CT
- [ ] Robotic navigation without intraop CT using alternate registration methods

Other: [ ] ____________________

15. If any, What barriers do you have to adopting instrumentation placement adjuntcs (free text answer, suggested answers include: *

Tick all that apply.

- [ ] Cost of equipment
- [ ] Limited availability of trained staff to run equipment
- [ ] Limited availability of trained staff to service equipment
- [ ] Incompatibility with available hardware
- [ ] Inexperience with fluoroscopy mediated methods
- [ ] Inexperience with manual stereotactic computer navigation methods
- [ ] Inexperience with robotic mediated methods

Other: [ ] ____________________

This content is neither created nor endorsed by Google.

Google Forms
