# Supplementary material for: Prevalence of spine surgery navigation techniques and availability in Africa: A cross-sectional study
Source: Ann Med Surg (Lond). 2021 Jul 29;68:102637. doi: 10.1016/j.amsu.2021.102637 (PMC8346523; doi:10.1016/j.amsu.2021.102637)
Supplement: Multimedia component 3 [file mmc3.docx]

**Supplemental Material – 95% Confidence Intervals**

**Table A.1. Sociodemographic characteristics**

| **Characteristics** | **Percentage (95% CI)** |
| --- | --- |
| Sex  Male  Female  Region  Northern Africa  Western Africa  Central Africa  Eastern Africa  Southern Africa  Specialty  Neurosurgery  Orthopedics  Practice  Public academic  Private  Public non-academic  Military  Academic level  Resident  Consultant  Fellow | 84.1 (77.0-90.3)  15.9 (9.7-23.0)    46.4 (37.5-56.2)  21.4 (14.3-29.5)  10.7 (5.4-17.0)  9.8 (4.5-16.0)  11.6 (6.3-17.9)  86.7 (79.5-92.9)  13.3 (7.1-20.5)    74.3 (65.2-82.1)  19.5 (11.6-27.7)  3.3 (7.2-19.6)  8.8 (3.6-14.3)    44.2 (36.3-53.1)  41.6 (32.7-50.4)  14.2 (8.0-21.2) |

**Table A.2. Availability of spine surgery instrumentation navigation techniques**

| **Navigation technique** | **Percentage (95% CI)** |
| --- | --- |
| Overall  Freehand  Fluoroscopy  Stereotactic without intraoperative CT  Stereotactic with intraoperative CT  Robotic without intraoperative CT  Robotic with intraoperative CT  Regional  *Northern Africa*  Freehand  Fluoroscopy  Stereotactic without intraoperative CT  Stereotactic with intraoperative CT  Robotic without intraoperative CT  Robotic with intraoperative CT  *Western Africa*  Freehand  Fluoroscopy  Stereotactic without intraoperative CT  Stereotactic with intraoperative CT  Robotic without intraoperative CT  Robotic with intraoperative CT  *Central Africa*  Freehand  Fluoroscopy  Stereotactic without intraoperative CT  Stereotactic with intraoperative CT  Robotic without intraoperative CT  Robotic with intraoperative CT  *Eastern Africa*  Freehand  Fluoroscopy  Stereotactic without intraoperative CT  Stereotactic with intraoperative CT  Robotic without intraoperative CT  Robotic with intraoperative CT  *Southern Africa*  Freehand  Fluoroscopy  Stereotactic without intraoperative CT  Stereotactic with intraoperative CT  Robotic without intraoperative CT  Robotic with intraoperative CT  Occipital axial  Freehand  Fluoroscopy  Stereotactic without intraoperative CT  Stereotactic with intraoperative CT  Robotic without intraoperative CT  Robotic with intraoperative CT  Subaxial  Freehand  Fluoroscopy  Stereotactic without intraoperative CT  Stereotactic with intraoperative CT  Robotic without intraoperative CT  Robotic with intraoperative CT  Thoracic  Freehand  Fluoroscopy  Stereotactic without intraoperative CT  Stereotactic with intraoperative CT  Robotic without intraoperative CT  Robotic with intraoperative CT  Lumbosacral  Freehand  Fluoroscopy  Stereotactic without intraoperative CT  Stereotactic with intraoperative CT  Robotic without intraoperative CT  Robotic with intraoperative CT  Pelvic  Freehand  Fluoroscopy  Stereotactic without intraoperative CT  Stereotactic with intraoperative CT  Robotic without intraoperative CT  Robotic with intraoperative CT | 56.3 (46.4-65.2)  96.4 (92.9-99.9)  32.1 (23.2-40.2)  8.9 (4.5-14.3)  6.3 (2.7-11.6)  29.5 (20.5-37.5)  50.9 (41.6-60.2)  98.1 (95.6-100)  49.1 (39.8-58.4)  0  0  49.1 (39.8-58.4)  87.5 (88.4-93.6)  100  29.2 (20.8-37.6)  33.3 (24.6-42.0)  29.2 (20.8-37.6)  29.2 (20.8-37.6)  91.7 (86.6-96.8)  100  0  0  0  0  18.2 (11.1-25.4)  81.8 (74.7-89.0)  18.2 (11.1-25.4)  18.2 (11.1-25.4)  0  0  15.4 (8.7-22.1)  92.3 (87.4-97.2)  7.7 (2.8-12.6)  0  0  0  55.4 (45.5-64.3)  95.5 (91.1-99.1)  8.0 (3.6-13.4)  8.9 (3.6-14.3)  6.3 (2.7-10.7)  6.3 (2.7-10.7)  55.4 (45.4-64.3)  95.5 (91.1-99.1)  8.9 (3.6-14.3)  8.9 (3.6-14.3)  0  6.3 (2.7-10.7)  55.4 (45.5-64.3)  95.5 (91.1-99.1)  8.9 (3.6-14.3)  8.9 (3.6-14.3)  6.3 (2.7-10.7)  6.3 (2.7-10.7)  56.3 (46.5-65.2)  94.6 (90.2-98.2)  9.8 (4.5-15.2)  8.0 (3.6-13.4)  6.3 (2.7-10.7)  6.3 (2.7-10.7)  56.3 (46.5-65.2)  92.0 (86.6-96.4)  8.0 (3.6-13.4)  9.8 (4.5-15.2)  6.3 (2.7-10.7)  6.3 (2.7-10.7) |

**Table A.3. Barriers to spine surgery instrumentation navigation techniques**

| **Barriers** | **Percentage (95% CI)** |
| --- | --- |
| Overall  Cost of equipment  Lack of trained staff to service the equipment  Lack of trained staff to run the equipment  Hardware incompatibility  Inexperience  Regional  *Northern Africa*  Cost of equipment  Lack of trained staff to service the equipment  Lack of trained staff to run the equipment  Hardware incompatibility  Inexperience  *Western Africa*  Cost of equipment  Lack of trained staff to service the equipment  Lack of trained staff to run the equipment  Hardware incompatibility  Inexperience  *Central Africa*  Cost of equipment  Lack of trained staff to service the equipment  Lack of trained staff to run the equipment  Hardware incompatibility  Inexperience  *Eastern Africa*  Cost of equipment  Lack of trained staff to service the equipment  Lack of trained staff to run the equipment  Hardware incompatibility  Inexperience  *Southern Africa*  Cost of equipment  Lack of trained staff to service the equipment  Lack of trained staff to run the equipment  Hardware incompatibility  Inexperience | 94.7 (92.0-99.1)  63.7 (55.4-73.2)  60.2 (51.8-69.6)  40.7 (32.1-50.9)  39.8 (31.3-50.0)  94.2 (89.9-98.5)  56.6 (47.4-65.8)  54.7 (45.5-63.9)  58.5 (49.4-67.6)  69.8 (61.3-78.3)  95.8 (92.1-99.5)  12.5 (6.4-18.6)  12.5 (6.4-18.6)  29.2 (20.8-37.6)  33.3 (24.6-42.0)  91.7 (86.6-96.8)  25.0 (17.0-33.0)  25.0 (17.0-33.0)  75.0 (67.0-83.0)  50.0 (40.7-59.3)  100  63.6 (54.7-72.5)  63.6 (54.7-72.5)  81.8 (74.7-88.9)  81.8 (74.7-88.9)  100  23.1 (15.3-30.9)  23.1 (15.3-30.9)  92.3 (87.4-97.2)  92.3 (87.4-97.2) |
